# Bioactive decellularized extracellular matrix-based hydrogel supports human adipose tissue-derived stem cell maintenance and fibrocartilage phenotype

**DOI:** 10.3389/fbioe.2023.1304030

**Published:** 2024-01-08

**Authors:** Raphaella Josino, Marco Augusto Stimamiglio

**Affiliations:** Laboratory of Stem Cells Basic Biology, Carlos Chagas Institute—FIOCRUZ Paraná, Curitiba, Brazil

**Keywords:** decellularized extracellular matrix, mesenchymal stem cells, regenerative medicine, tissue engineering, cartilage

## Abstract

Articular cartilage is a highly specialized tissue able to tolerate physical stress. However, its capacity for restoration is restricted, and injuries to the cartilage do not recover spontaneously. Interest in mesenchymal stem cells derived from human adipose tissue (hASCs) is growing due to their potential to improve tissue healing and recovery. Decellularized extracellular matrix (dECM)-based hydrogels combined with hASCs could serve as an interface for studying behavior and differentiation properties in a cartilage microenvironment. In the present study, we described the behavior of hASCs cultured in a commercial dECM MatriXpec™. The structural microtopography of MatriXpec™ was analyzed by scanning electron micrography, and its protein composition was accessed by mass spectrometry. The protein composition of MatriXpec™ is mainly represented by collagen proteins, building its fibrous ultrastructure. hASCs were cultured three-dimensionally (3D) on MatriXpec™ to perform cell viability, growth, and cartilage differentiation analysis. We showed that MatriXpec™ could be loaded with hASCs and that it supports cell maintenance for several days. We observed that the three-dimensional ultrastructure of the biomaterial is composed of nanofibers, and its protein composition reflects the tissue from which it was harvested. Finally, we showed that the molecular cues from the hydrogel are biologically active as these influence cell behavior and differentiation phenotype, increasing the expression of fibrocartilage-related genes such as SOX9, COL1, COL10, and MMP13. MatriXpec™ hydrogel can be used as an interface for 3D hASCs culture studies as it maintains cell viability and supports its differentiation process.

## 1 Introduction

Cartilage is a connective tissue characterized by a cellular component immersed in the extracellular matrix (ECM). It can be classified into three subtypes based on differences in ECM composition and organization: hyaline, elastic, and fibrocartilage (Armiento et al., 2019). Human mobility is ensured by a complex skeletal system, mainly composed of cartilage and bones (Gahunia and Pritzker, 2012; Thomeer et al., 2022). The bone joint preserves the mechanical competence of the skeletal system by providing a gliding surface free of intense friction. This ability to absorb the mechanical impact that reaches the joints is due to hyaline-type articular cartilage (Gomoll and Minas, 2014). Moreover, the knee joints contain menisci, which are fibrocartilage structures that provide support against the mechanical overload associated with the tibiofemoral joint (Chen et al., 2017).

Injuries to articular cartilaginous tissues (articular cartilage and the menisci) damage the ECM, resulting mainly in type I and II collagen fiber failure, depletion of proteoglycans and glycosaminoglycans (GAGs), and a substantial loss of chondrocytes (Rahmati et al., 2017). The result is a reduction in the viscoelasticity of the cartilage tissue and an inability of a joint system to absorb shock, causing pain, swelling, and mobility loss. Furthermore, long-term damage may lead to degenerative joint changes like articular cartilage and meniscus degeneration, joint space narrowing, and a high risk of developing osteoarthritis (OA), the most common form of degenerative joint disease (Hunter et al., 2014; Rahmati et al., 2017).

It is impossible to recover spontaneously from these injuries. In an attempt to reverse the pain and mobility loss experienced by patients, pharmacological and surgical interventions have been employed. However, none of these interventions can restore tissue structure and functionality (Anderson et al., 2014). Considering the need for effective clinical strategies to restore the injured tissue features, there is a demand for regenerative medicine and tissue engineering approaches to reverse damage following injuries to the articular cartilage (Richter et al., 2016).

In this context, therapies based on the use of human cells have been used as a strategy to restore damaged tissues (Richardson et al., 2016; Miana and Prieto González, 2018). Autologous chondrocyte implantation (ACI) was the first cell therapy used to repair injuries to articular cartilaginous tissue. It consists of collecting a healthy and unloaded portion of the patient’s cartilage, isolating and expanding the chondrocytes *in vitro*, and re-implanting them into the injured area (Brittberg et al., 1994). However, there are some disadvantages associated with this therapeutic approach, such as the need for two surgeries—the first one to remove the healthy cartilage and the second one to transplant the cultured chondrocytes, as well as the low functionality and quality of the neo-synthesized ECM (Horas et al., 2003). Currently, the microfracture technique is the first-line treatment for articular chondral injuries. This approach involves performing microfractures in the subchondral bone tissue to stimulate the migration of mesenchymal stem cells (MSCs) to the site of the cartilaginous injury (Makris et al., 2015). For regeneration of meniscus fibrocartilage, local injections with MSCs have been employed (Itose et al., 2022). For both strategies, a major challenge is to keep the cells at the exact injury site to which MSCs exert their healing effects, as applied cells tend to disperse throughout the tissue, and the injured tissue is not able to benefit from their therapeutic properties (W. Zhang et al., 2021).

MSCs have sparked clinical interest in the treatment of many conditions and diseases (Maldonado et al., 2023). Initially, the therapeutic effects exerted by MSCs were attributed exclusively to their ability to migrate and enter damaged tissues, replacing dead cells. However, other therapeutic benefits of MSCs for protecting and repairing damaged tissues have been discovered, as have their paracrine anti-apoptotic, scar-inhibitory, angiogenesis-stimulating, and mitogenic effects for tissue intrinsic progenitors (Caplan, 2017). Moreover, MSCs are producers of extracellular vesicles, cytokines, and growth factors (secretome) that modulate both the innate and adaptive immune systems (Lee et al., 2023). This immunoregulatory role is supported by *in vitro*, *in vivo*, and clinical studies, which reveal a complex network of interactions between immune system cells and MSCs (Naji et al., 2019). As a stem cell population, human adipose-derived stem cells (hASC) can maintain self-renewal and multidifferentiation potential, including for chondrogenic phenotype. Although there are different sources for obtaining mesenchymal stem cells (MSCs), the use of adipose-derived MSCs has advantages over other sources, such as their greater availability for collection (Naji et al., 2019).

Studies have been performed on combining MSCs and biomaterials for the tissue-derived decellularized extracellular matrix (dECM) to restore damaged tissue functionality (X. Zhang et al., 2022). Due to the high similarity with the physiological tissue and the specific architecture and composition of the organ, biomaterials based on dECM have low cytotoxicity and are immunocompatible (Saldin et al., 2017). Indeed, *in vivo* studies have already demonstrated that this similarity with the native tissue makes natural biomaterials highly advantageous for biomedical applications than synthetic biomaterials (Yao et al., 2017; Taylor et al., 2018). dECM-based hydrogels are produced from decellularized tissues, and these can be used for *in situ* application, three-dimensional cell culture, and delivery of molecules and drugs (Radhakrishnan et al., 2017; Shakouri-Motlagh et al., 2017; Yao et al., 2019).

The present study builds on previous reports that discuss the use of dECM-based hydrogels for three-dimensional cell culture models (Taylor et al., 2018; Isaeva et al., 2022; Liu et al., 2022). We hypothesized that commercial MatriXpec™ hydrogel derived from decellularized cartilage tissue brings a favorable environment for stem cell growth and maintenance. We also explored the influence of MatriXpec™ three-dimensional environment on the viability, proliferation, and differentiation of human adipose-derived stem cells (hASCs).

## 2 Materials and methods

### 2.1 hASCs isolation and culture

This study followed the Ethical Issues for Research Involving Human Subjects principles and was approved by the Research Ethics Committee of Fundação Oswaldo Cruz, Brazil (CAAE 48374715.8.0000.5248). The cells used in this study were harvested from adipose tissue derived from liposuction that would otherwise be discarded, therefore does not present ethical issues or risks for the donor, who provided written informed consent. Cells were obtained according to a previously described protocol (Silva et al., 2012). Briefly, fragments of subcutaneous adipose tissue were washed with phosphate-buffered saline (PBS) and minced into small fragments before being digested with 1 mg/mL type I collagenase (Invitrogen^®^, Grand Island, NY, United States). The digested tissue was passed through a filter, washed with PBS, and centrifuged. The cell pellet was resuspended in DMEM-F12 medium (Invitrogen^®^) supplemented with 10% fetal bovine serum (FBS) for plating on culture flasks. The stem cells’ identity was later confirmed through flow cytometry immunophenotyping for classic positive (CD90, CD105, CD73, and CD140b) and negative (CD19, CD11b, HLA-DR, CD45, CD34, and CD31) membrane markers (Horinouchi et al., 2020) and evaluation of adipogenic, osteogenic and chondrogenic differentiation potential as previous described (Dominici et al., 2006). For subsequent experiments, hASCs were cultivated in Dulbbecco’s modified Eagle’s medium (DMEM) (Gibco Invitrogen^®^, Carlsbad, California, United States), 4 mM L-glutamine (Gibco Invitrogen^®^, Carlsbad, California, United States), 100 U/mL penicillin and 100 ug/mL streptomycin (Sigma-Aldrich, Saint Louis, MO, United States) at 37°C with 95% humidity and 5% CO_2_. Experiments were conducted on the cells between passages 4 and 6.

### 2.2 MatriXpec preparation

MatriXpec™ was purchased from TissueLabs Company (MatriXpec Thermo, Ref.#MXPC-CA). The hydrogel was fabricated from cartilage dECM as previously described (Liguori et al., 2020). Briefly, porcine tendons (16-week-old pigs) were dissected and isolated. The collected tissue was washed in PBS, triturated into small fragments, and incubated in 0.05% trypsin in PBS. After incubation, the tissue was washed with PBS and passed through cycles of freezing and treatment with a detergent solution (sodium dodecyl sulfate—SDS) in PBS. After detergent treatment and washing, tissue was incubated with DNAse solution, washed overnight with 70% ethanol, and stored at 4°C in 1% penicillin/streptomycin in sterile PBS. The final product of this protocol is denoted as dECM MatriXpec™ and was used to cover the culture wells prior to hASCs seed. The first step in the plating process of the MatriXpec™ involves neutralizing the dECM using the neutralizing buffer provided by the manufacturer. The required amount of neutralizing buffer to prepare 3D hydrogels corresponds to 1/9 of the total desired volume of dECM solution. The tube containing the solution was gently shaken to avoid bubble formation, and after homogenization, the pH was measured, with the result indicating that it was almost neutral. After plating, it was incubated at 37°C with 95% humidity and 5% CO_2_ for 1 h to promote hydrogel gelation. The resulting 3D hydrogel was used in the experiments. Batch number of MatriXpec^TM^ and neutralizing buffer: MXPCCA10221301.

### 2.3 Proteomic characterization of the MatriXpec

For Western Blotting analysis, the protein concentration of the MatriXpec™ hydrogel was quantified using the Qubit™ Protein Assay kit (Molecular Probes; Invitrogen Life Technologies^®^, Carlsbad, CA, United States), according to the manufacturer’s instructions. Next, 20 µg of protein was loaded into a 13% SDS gel, separated by electrophoresis, and transferred to a 0.45 µm nitrocellulose membrane (BioRadTM, Hercules, CA, United States) using a Trans-BlotTM SD Semi-dry Transfer Cell. The membrane was blocked with 5% milk for 1 h at room temperature and incubated overnight with anti-COL2A1 antibody 1:1,000 (Abcam, Cambridge, United States). Finally, the membrane was incubated for 1 h with IgG anti-mouse antibody 1:15,000 (Odyssey IRDyeTM, LI-COR Biosciences, Lincoln, NE, United States). The LI-COR Odyssey (BioAgilytixTM, Durham, NC, United States) was employed to acquire and analyze images.

Proteins were digested using high sequence grade modified trypsin at a concentration of 1:50 at 37°C for approximately 20 h for mass spectrometry analysis. After sample digestion, all reaction products were acidified to stop proteolysis. The samples were centrifuged to remove insoluble materials. Peptides were desalted using a Pierce^®^ C18 spin column (Thermo Scientific™, Waltham, MA, United States), as described by Rappsilber and others (RAPPSILBER; ISHIHAMA; MANN, 2003). The peptides were analyzed using the Orbitrap Fusion Lumos™ mass spectrometer (Thermo Scientific™, Waltham, MA, United States). Each peptide mixture was suspended in 0.1% formic acid for data acquisition in Orbitrap. The peptide mixture was separated by chromatography using Ultimate 3000 HPLC equipment (Thermo Scientific™, Waltham, MA, United States). The spectrometer was set in data-dependent acquisition mode to automatically switch between full scan MS and MS/MS acquisition with 20-s dynamic exclusion. Survey scans (350–2000 m/z) were acquired on the Orbitrap system with a resolution of 60,000 at 200 m/z. Mass spectrometer scan functions were controlled by the Xcalibur v4.1 data system (Thermo Scientific™, Waltham, MA, United States).

### 2.4 Cell growth curve

Cell nuclei were counted after 2, 5, 10, 14, and 21 days of cell culture to evaluate the cell growth dynamics on MatriXpec™. Additionally, on days 2, 5, and 20, the DNA of the cells was extracted using a homebrew DNA extracting protocol using Proteinase K enzymatic digestion and quantified using a NanoDrop One Microvolume Spectrophotometer (Thermo Scientific™, Waltham, MA, United States).

### 2.5 Cell viability analysis

Lactate dehydrogenase enzyme (LDH) release was quantified to assess cell viability, using the CytoTox 96^®^ Non-Radioactive Cytotoxicity Assay Kit (Promega, Madison, WI, United States). The hASCs were cultured in the TCP or MatriXpec™ (1.5 × 103 cells/well), and after 2 and 5 days, the culture media was collected and prepared for analysis according to the manufacturer’s protocol. The LDH concentration was obtained by measuring supernatant absorbance at 490 nm using a Synergy H1 Hybrid Multiplate Microplate Reader (Biotek^®^, Winooski, VT, United States). The LIVE/DEAD Viability/Cytotoxicity Kit (Thermo Scientific™) was also used for qualitative analysis. The staining solution, containing 0.6 µM ethidium homodimer-1 and 0.4 mM calcein-AM, was incubated for 30 min at TCP or MatriXpec™ cell culture conditions and then observed under a microscope. The samples were analyzed at ×20 magnification in an AF6000 inverted fluorescence microscope (Leica Microsystems, Wetzlar, Germany).

### 2.6 Cell morphology examination

To evaluate changes in cell morphology in response to culture microenvironment, 1.5 × 103 cells/well were plated on TCP or MatriXpec™. After 7, 14, and 21 days, hASCs were fixed with 4% paraformaldehyde (PFA) and incubated with rabbit anti-β-Tubulin antibody 1:100 (ab15568, Abcam, Cambridge, United States) diluted in 1% PBS/BSA (bovine resum albumin) and 0.5% Triton X-100, overnight at 4°C under gentle shake. The samples were washed and incubated with secondary anti-rabbit Alexa Fluor 488 1:800 (A21206, Invitrogen Life Technologies^®^, Carlsbad, CA, United States) diluted in a 1% PBS/BSA 0.1% Triton X-100 solution for 3 h. Following the incubation period, samples were washed and stained using DAPI. The samples were analyzed at ×20 magnification in an AF6000 inverted fluorescence microscope (Leica Microsystems, Wetzlar, Germany).

Scanning Electron Microscopy (SEM) was performed to characterize both the MatriXpec™ behavior in the cell culture plate after gelation and the morphology of hASCs seeded above the 3D hydrogel. The SEM analysis was performed before cell seeding for hydrogel three-dimensional structure and organization analysis, while for the cell adhesion analysis, hASCs were cultured on the MatriXpec™ surface for 7 days. The samples were fixed with 2.5% glutaraldehyde (in 0.1 M sodium cacodylate buffer) for 1 h under room temperature. After fixation, samples were washed with a 0.1 M sodium cacodylate buffer and incubated in a solution of 1% osmium tetroxide (in 0.1 M sodium cacodylate buffer) for 40 min. Samples were dehydrated using growing ethanol concentrations (30¬–100%), then submitted to critical point drying, coated with gold, and analyzed using the scanning electron microscope (JEOL JSM6010 PLUS-LA, JEOL Ltd., Tokyo, Japan).

### 2.7 Chondrogenic differentiation

To evaluate the influence of MatriXpec™ on hASCs chondrogenic fate, 1.5 × 103 cells were seeded in 24-well plates. After 3 days of culture, the cells were induced for chondrogenic differentiation for a period of 10 and 21 days using Human Mesenchymal Stem Cell (hMSC) Chondrogenic Differentiation Medium BulletKit™ (Lonza Bioscience^®^, Walkersville, United States). The chondrogenic medium was used together with the SingleQuots™ (Lonza Bioscience^®^, Walkersville, United States): dexamethasone, ascorbic acid, insulin-transferrin-selenium supplement, gentamicin/amphotericin, sodium pyruvate, proline, and L-glutamine. The differentiation medium was renewed twice weekly for 10 and 21 days. Before all medium changes of the hASCs differentiation cultures, the medium was supplemented with 10 ng/mL of TFG-β3 (Lonza Bioscience^®^, Walkersville, United States). Cartilage differentiation of the hASCs after 10 and 21 days of culture was assessed by 1) Safranin O staining, 2) quantification of GAGs through the DMMB assay, and 3) expression of chondrogenic genes using real-time quantitative polymerase chain reaction (RT-qPCR).

### 2.8 Safranin-O staining

The hASCs of induced (chondro) and non-induced (control) experimental conditions of TCP or MatriXpec™ plating were initially fixed with 4% PFA and then washed with PBS. Cells were stained with 0.1% Safranin O solution (Sigma-Aldrich, St. Louis, MO, United States) diluted in deionized water for 30 min at room temperature. After the removal of the Safranin O solution, samples were washed. The plates were analyzed at ×20 magnification in a DMi8 inverted fluorescence microscope (Leica Microsystems, Wetzlar, Germany).

### 2.9 GAG quantification

The hASCs cultured in induced (chondro) and non-induced (control) in TCP or MatriXpec™ surface were analyzed for their potential to deposit GAGs through GAG quantification using 1.9-dimethyl-methylene blue (DMMB) dye. Following the differentiation protocol, cells were incubated overnight at 65°C with a papain digestion solution (100 mM sodium phosphate buffer, 10 mM EDTA, 10 mM L-cysteine, and 0.125 mg/mL papain). Following incubation, the samples were centrifuged at 10,000 g for 10 min, and the supernatant was recovered for DNA and GAG quantification. The DNA was quantified using the Qubit™ dsDNA HS Assay kit (Molecular Probes; Invitrogen Life Technologies^®^, Carlsbad, CA, United States) according to the manufacturer’s instructions. For GAG quantification, a solution of 0.16% DMMB (Sigma-Aldrich, St. Louis, MO, United States), 0.24% NaCl, 0.30% glycine, and 10 mM HCl was prepared and added to the supernatant containing the samples of extracted GAG. The absorbance of the resulting solutions was measured at 520 nm using a Synergy H1 Hybrid Multiplate Microplate Reader (Biotek^®^, Winooski, VT, United States). The GAG content was selected to fit into the range of a standard curve (sGAG reference standard; Biocolor, Northern Ireland, United Kingdom). GAG quantification results were normalized from DNA quantification for each sample.

### 2.10 RNA isolation and quantitative reverse transcriptase polymerase chain reaction

After 10 and 21 days of following the chondrogenic differentiation protocol, the RNA of the hASCs was extracted with TriReagent (Sigma-Aldrich, Saint Louis, MO, United States) and isolated with the Direct-zolTM RNA MiniPrep (Zymo Research, Tustin, CA, United States), according to manufacturer’s instructions. The yield and purity of RNA were evaluated using a NanoDrop™ One Microvolume UV-Vis Spectrophotometer (Thermo Scientific™, Waltham, MA, United States), and 500 ng per sample were used to synthesize cDNA, following ImProm-IITM Reverse Transcription System (Promega, Madison, WI, United States) kit instructions. The RT-qPCR samples were prepared according to the manufacturer’s instructions (GoTaq^®^ qPCR and RT-qPCR; Promega, Madison, WI, United States) and performed in technical triplicate, based on cell differentiation experiments from three distinct cell donors. The analyzed genes were: SOX9 (SRY-box transcription factor 9; forward primer 5′-AAG​AAC​AAG​CCG​CAC​GTC​AA-3′ and reverse primer 5′-CCG​TTC​TTC​ACC​GAC​TTC​CTC-3′); COL2A1 (collagen type II; forward primer 5′-CAT​CCC​ACC​CTC​TCA​CAG​TT-3′ and reverse primer 5′-GCC​TCT​GCC​TTG​ACC​CGA​AG-3′); ACAN (aggrecan; forwar primer 5′-CAC​TGT​TAC​CGC​CAC​TTC​CC and reverse primer 5′-GAC​GAT​GCT​GCT​CAG​GTG​TG); MMP13 (matrix metallopeptidase 13; forward primer 5′-CAT​GAG​TTC​GGC​CAC​TCC​TT-3′ and reverse primer 5′-CCT​CGG​AGA​CTG​GTA​ATG​GC-3′); COL10A1 (collagen type X; forward primer 5′-CCA​GCA​CGC​AGA​ATC​CAT​CTG​A-3′ and reverse primer 5′-CTT​GGT​GTT​GGG​TAG​TGG​GC-3′); COL1A1 (collagen type I; forward primer 5′-AGG​GCT​CCA​ACG​AGA​TCG​AGA​TCC​G-3′ and reverse primer 5′-TAC​AGG​AAG​CAG​ACA​GGG​CCA​ACG​TCG-3′); FMOD (fibromodulin; forward primer 5′-GGG​CAT​ACA​ACC​CTC​TGC​TT-3′ and reverse primer 5′-GTG​CTC​CCC​AGC​CAA​ACT​AT-3′) and RNAPOL2 (RNA polymerase II; forward primer 5′-TAC​CAC​GTC​ATC​TCC​TTT​GAT​GGC​T-3′ and reverse primer 5′-GTG​CGG​CTG​CTT​CCA​TAA-3′) which was used as an internal control. The data were obtained through a QuantStudioTM 5 Real-Time PCR System (Thermo Fisher, Waltham, MA, United States) and analyzed following the 2^−ΔΔCT^ using QuantStudioTM Design and Analysis Software v1.5.2.

### 2.11 Statistical analysis

GraphPad Prism v8.0 was used to perform statistical analysis. An unpaired student’s t-test was used to compare the two groups. An ordinary two-way ANOVA was used to compare multiple treatment groups. All data were expressed as the mean ± standard deviation (SD), and a *p*-value <0.05 was considered statistically significant.

## 3 Results

### 3.1 MatriXpec is composed of collagen proteins and has a fibrous ultrastructure

**FIGURE 1:**
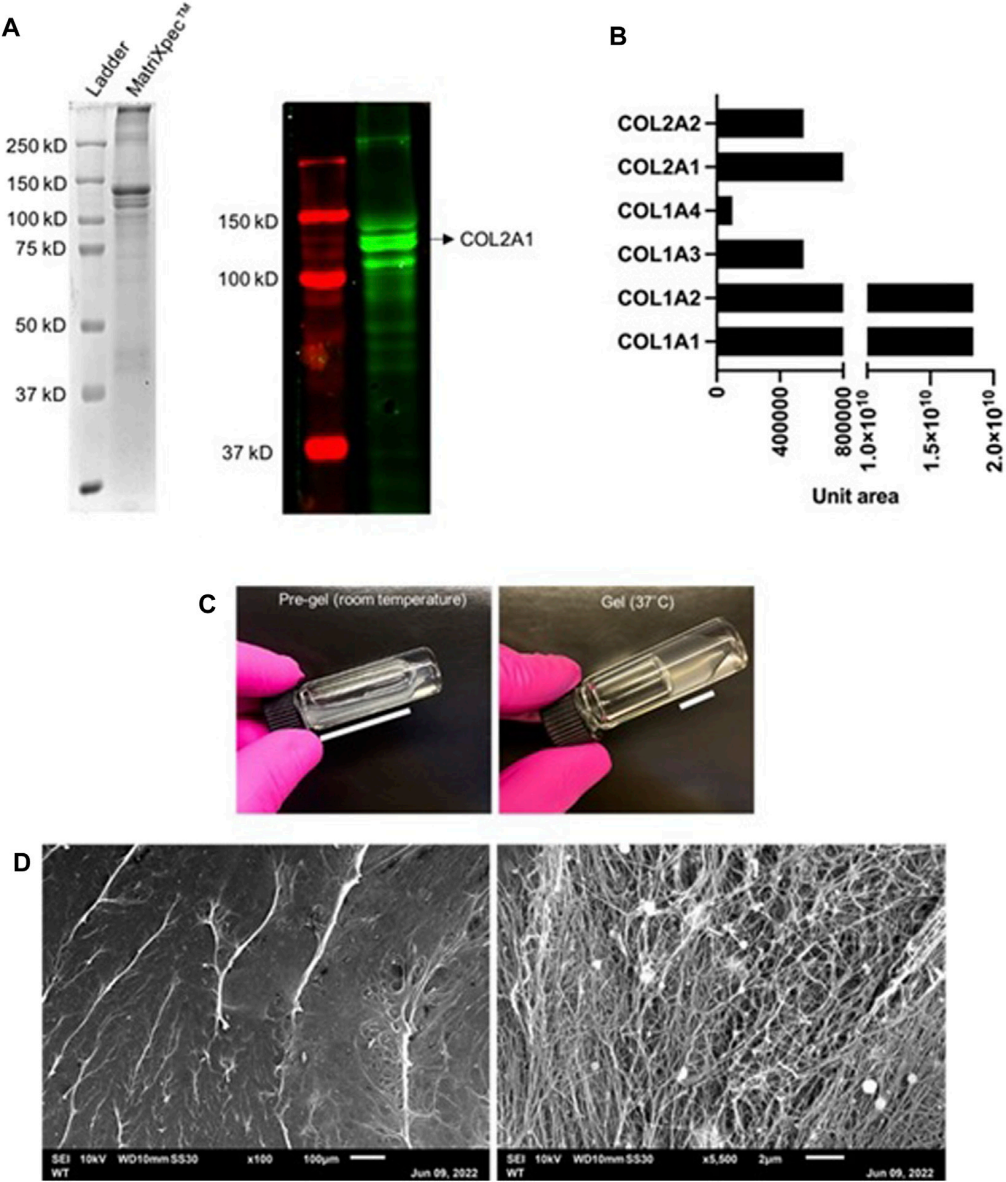
Protein profiling and surface ultrastructure of MatriXpec^TM^. **(A)** The overall protein profile was visualized in an SDS-PAGE followed by Western Blotting labeling for COL2. **(B)** Most abundant proteins detected by mass spectrometry in MatriXpec^TM^ hydrogel. **(C)** Gelation process of the biomaterial in response to 37°C temperature. **(D)** Visualization of MatriXpec^TM^ by SEM analysis.

Considering that type 2 collagen protein is abundant in native cartilaginous tissue, Western Blotting analysis confirmed the presence of COL2 in MatriXpec™ as the most prominent protein band with approximately 150 kD (Figure 1A). The total protein content of MatriXpec™ hydrogel analyzed by mass spectrometry revealed collagenous proteins as the most abundant in the biomaterial, mainly type 1 and 2 collagens, although type 6 and 9 collagens were also detected in the samples (Figure 1B). MatriXpec™ is a thermoresponsive hydrogel that changes its physical state from liquid to solid when exposed to a temperature of 37°C (Figure 1C), a process known as gelation. After gelation, SEM was performed to monitor the MatriXpec™ surface appearance. Surface features are visualized in Figure 1D, demonstrating that the biomaterial can cover the culture well and has a fibrillar ultrastructure, forming a porous scaffold rich in three-dimensional information.

### 3.2 MatriXpec supports hASCs viability and growth

**FIGURE 2:**
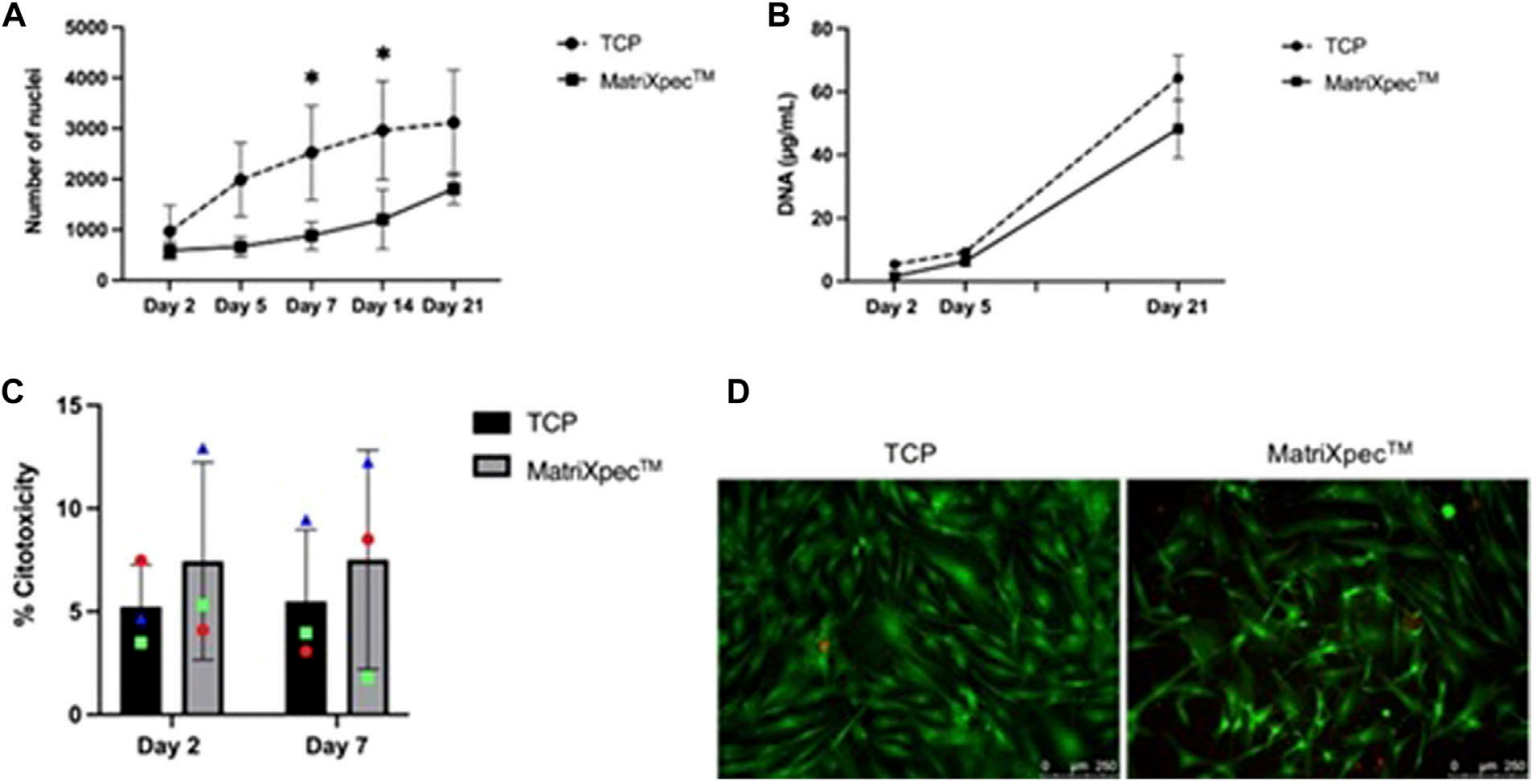
Biocompatibility of MatriXpec^TM^ with hASCs. The dynamics of cell growth and maintenance were assessed by different methodologies. **(A)** Growth dynamics of cells seeded in TCP and MatriXpec^TM^ after 2, 5, 7, 14, and 21 days of cell culture analyzed through nuclei count. **(B)** Growth dynamics of cells seeded in TCP and MatriXpec^TM^ at days 2, 5, and 21 analyzed through DNA extraction and quantification. **(C)** Percentage of cell death in TCP and MatriXpec^TM^ after 2 and 7 days of cell culture evaluated by LDH assay; and **(D)** LIVE/DEAD staining of TCP and MatriXpec^TM^ cell cultures after 7 days. Data are represented as the mean ± SD. Each colored symbol represents a different hASC donor. Two-way ANOVA was performed to compare TCP samples with MatriXpec^TM^ at different time points. *p < 0.05.

Initially, we investigated whether hASCs growth dynamics were altered in cell cultures of MatriXpec™ compared to standard TCP experiments. After 2, 5, and 21 days of cell culture, DNA quantification and nuclei count did not show a statistical difference between TCP and MatriXpec™, indicating a similar number of cells at these time points (Figure 2A). However, on days 7 and 14 of cell culture, there was a statistical difference in the number of nuclei between TCP and MatriXpec™. On day 7, an average of 2,523.53 was counted in TCP and 883.33 in MatriXpec™, while on day 14, an average of 2,963.62 was counted in TCP and 1,203.67 in MatriXpec™ (Figure 2B). Taken together, these results indicate that after the first week of hASCs culture, cell population growth is slower in MatriXpec™; however, by day 21, cells reach a growth plateau in both culture conditions.

The LDH release levels were measured to assess the viability of hASCs after 2 or 7 days of culture in MatriXpec™. hASCs were highly viable in MatriXpec™, as there was no statistical difference in cell death percentage between TCP (5.22% ± 2.04) and MatriXpec™ (5.49% ± 4.79) after 2 days of culture. After 7 days of cell culture, cell death levels were maintained in the TCP (7.44% ± 3.47) and MatriXpec™ (7.52% ± 5.31) cultures (Figure 2C). Therefore, cells were analyzed qualitatively after 7 days of culture using LIVE/DEAD assay, confirming that cell culture on MatriXpec™ did not affect hASCs viability (Figure 2D), with viable cells labeled in green and nuclei of dead cells stained in red.

### 3.3 Cell culture in MatriXpec alters hASCs morphology and extracellular matrix deposition

**FIGURE 3:**
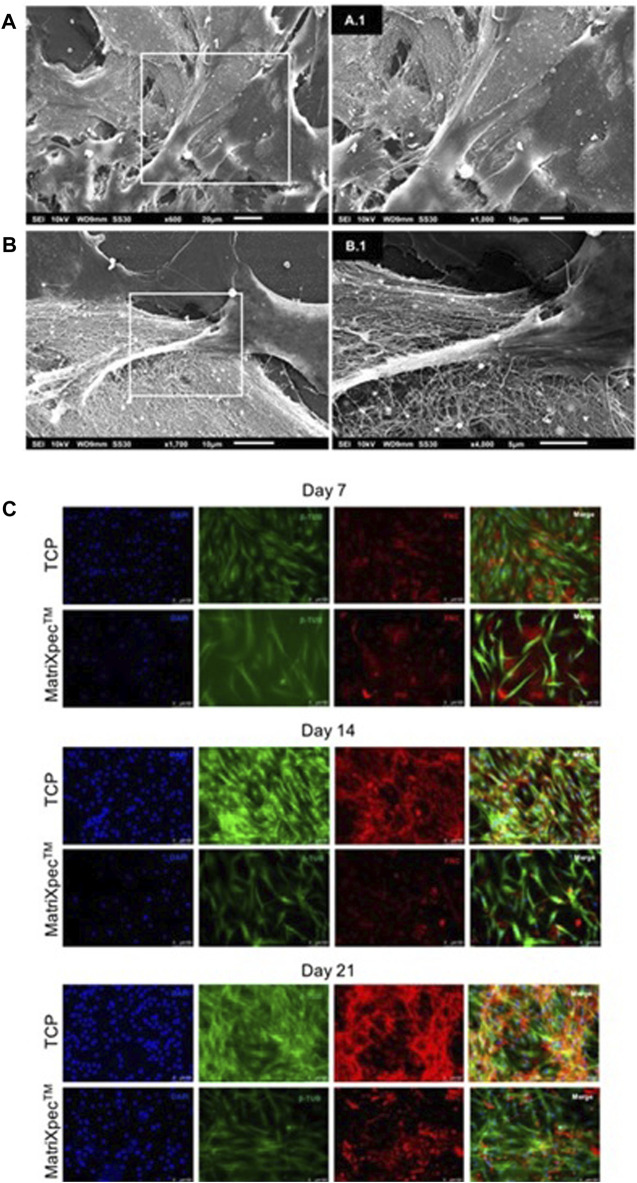
hASCs morphology analysis. Cell morphology was characterized by SEM analysis, β-Tubulin immunostaining (green labeling), and Fibronectin immunostaining (red labeling). **(A–A.1)** Cells adhered to MatriXpec^TM^ after 7 days of cell culture. **(B–B.1)** Cell membrane protrusions in response to biomaterial interaction. **(C)** Immunolabelling showing hASCs morphology and extracellular matrix deposition on days 7, 14, and 21 of cell culture.

The interaction between hASCs and MatriXpec™ was demonstrated by SEM analysis after 7 days of cell culture. When seeded in MatriXpec™, hASCs interact with the fibrous structure and spread over the hydrogel surface (Figures 3A–A.1). The hASCs showed numerous membrane protrusions, possibly associated with biomaterial interactions and cell migration (Figures 3B–B.1).

We performed immunofluorescence analysis to better access cell morphology in response to 3D culture on MatriXpec™. The cytoskeleton of hASCs was labeled with an anti-β-tubulin antibody, and the ECM was evidenced by labeling for fibronectin protein. After 7 days of cell culture, we observed that the hASCs initially seeded on the biomaterial surface were in different focal planes of the hydrogel, demonstrating the tendency of the cells to leave the surface and colonize the interior of MatriXpec™ (Figure 3C). This pattern of cell morphology on 3D culture was confirmed on days 14 and 21 of cell culture. hASCs cultivated in MatriXpec™ adopted a fusiform morphology in response to the 3D microenvironment, while the cells in TCP experiments adopted a spreading and flat morphology. Furthermore, the pattern of ECM deposition by the 3D cell cultures drew our attention. Cell cultures in MatriXpec™ showed a diffuse pattern of ECM deposition, compared to hASCs cultured in TCP, possibly associated with the presence of hASCs in different focal planes of the hydrogel (Figure 3C).

### 3.4 Culture of hASCs in MatriXpec favors GAG production and fibrocartilage differentiation

**FIGURE 4:**
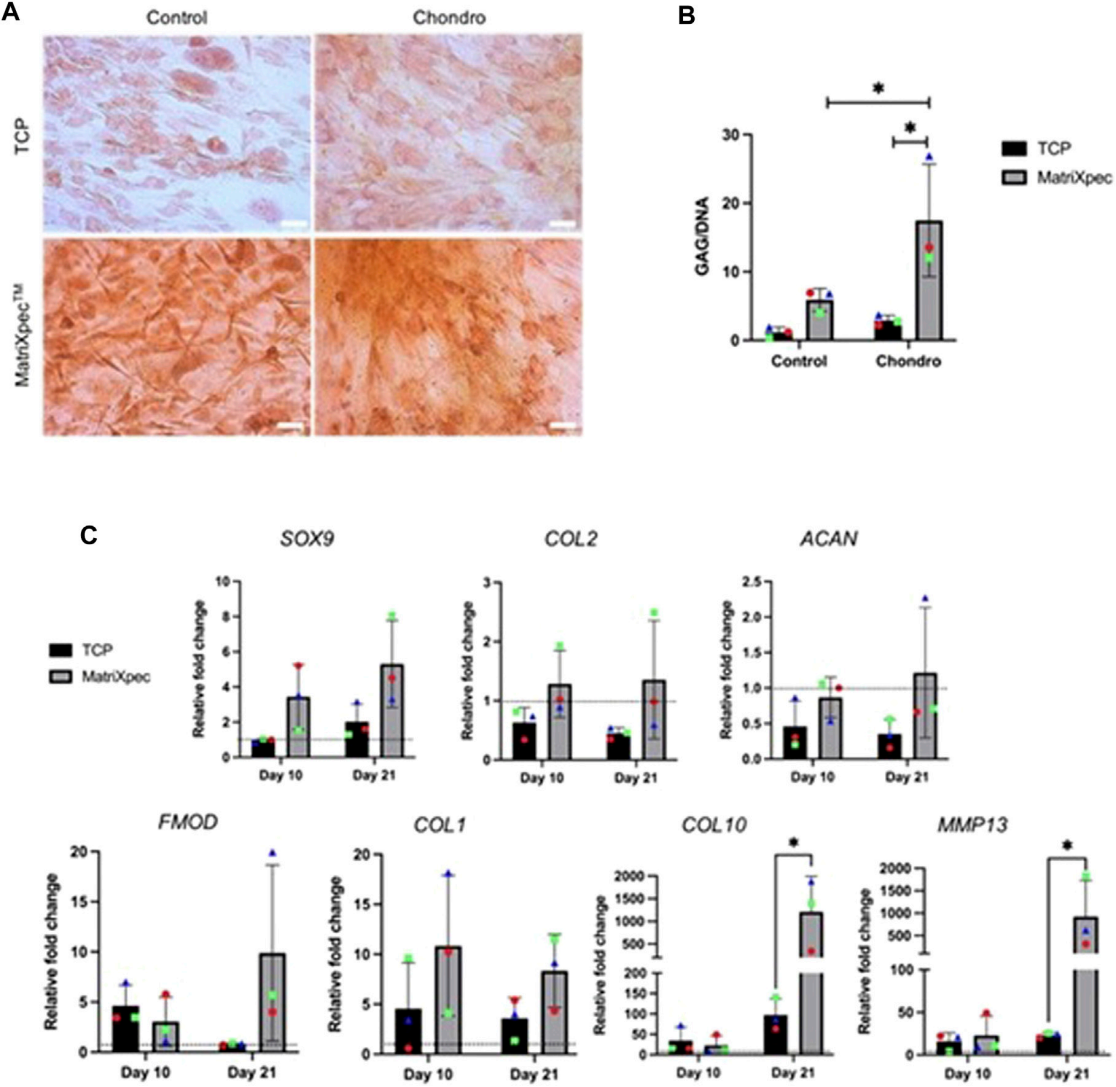
Chondrogenic differentiation analysis. Chondrogenic differentiation from hASCs cultured on TCP or MatriXpec^TM^ was evaluated by the presence of GAGs and markers associated with chondrogenic differentiation. **(A)** Safranin O staining (in orange) of hASCs induced (Chondro) or non-induced (Control) for chondrogenic differentiation after 21 days of cell culture. **(B)** Quantification of GAGs production after a 21-day period of chondrogenic differentiation (Chondro) or control experiments. **(C)** mRNA expression levels of SOX9, COL2A1, ACAN, FMOD, COL1A1, COL10, and MMP13 after 21 days of chondrogenic induction. Data are represented as the mean ± SD and were compared to the non-induced group (Control), represented by the dashed line in each graph. Each colored symbol represents a different hASC donor. Two-way ANOVA was performed to compare each treatment group. *p < 0.05.

Safranin O staining was carried out after 21 days of cell culture to analyze the overall effect of MatriXpec™ in hASCs chondrogenic differentiation, aiming to label deposited GAGs. We observed a higher GAG deposition in chondrogenic differentiation (Chondro) induction compared to control, both in TCP and MatriXpec™ conditions, with more pronounced staining in the MatriXpec™ condition. Furthermore, we showed that even under control conditions (hASCs cultivated with maintenance media), cells deposit GAGs when cultivated in MatriXpec™, suggesting that cell culture on the biomaterial alone is sufficient to induce GAG deposition by hASCs (Figure 4A). Quantification of GAG production through DMMB dye confirmed these results. The GAG quantification assay revealed higher GAG deposition by hASCs cultivated in a chondrogenic medium in MatriXpec™, compared with standard cell culture on TCP, with a statistical difference on day 21 (Figure 4B).

To better access the chondrogenic differentiation potential of hASCs seeded on MatriXpec™, the gene expression of chondrogenic markers SOX9, COL2A1, ACAN, FMOD, COL1A1, COL10, and MMP13 were evaluated. In general, when comparing MatriXpec™ to TCP cultures, a comparable state of differentiation could be achieved for most markers, except for COL10 and MMP13 genes, which showed a statistically significant upregulation in hASCs cultivated in MatriXpec™ (Figure 4C). This suggests that MatriXpec induces a higher expression of genes associated with the fibrocartilage phenotype. We performed encapsulation and micromass assays to evaluate cell culture and prove that the hASC are capable of differentiating in a chondrogenic phenotype (Supplementary Figure S1). Micromass analysis showed a cellularized core surrounded by a large amount of fibrous extracellular matrix, and no lacunae organization typical of the hyaline tissue, which is similar to the MatriXpec^TM^ culture results. These observations suggest that the chondrogenic differentiation protocol used in our work induces a fibrocartilage phenotype in hASC.

## 4 Discussion

Cartilage is a tissue with low intrinsic regeneration potential. To date, the most common treatment approaches to cartilage injuries are unsuitable for reversing cartilage defects, such as those that occur with OA. Many materials are currently being developed to treat cartilage injuries, and natural biomaterials have achieved biomedically relevant results. In this study, MatriXpec™ hydrogel was used for 3D cell culture of MSCs derived from hASCs. As a dECM hydrogel, MatriXpec™ should contain growth factors, GAGs, as well as collagenous proteins, which provide a desired microenvironment for cultured hASCs comparable to that of cartilage native tissue. The decellularized composites can promote cell viability, proliferation, and differentiation (Li et al., 2019). Moreover, these composites can be used as functional scaffolds with minimal immunogenicity for cell transplantation in regenerative medicine approaches (Makris et al., 2015; Sutherland et al., 2015).

Previous work has detailed the decellularization techniques employed to produce dECM-based hydrogels, histologically and molecularly validating the decellularization process, characterizing the protein composition of the dECM, and finally, accessing its potential use for the cultivation of mature chondrocytes (Stone et al., 2021). We evaluated the microtopography of MatriXpec™ hydrogel through SEM, corroborating the results of previous work (Stone et al., 2021) regarding the ultrastructure of the biomaterial, which is organized in nanofibres. Zhang and others (2021) warn that the main components remaining in decellularized composites are elastin, collagen, fibronectin, and matricellular proteins that have interaction sites with ECM proteins (Zhang et al., 2021). In our study, the ECM-related proteins identified were collagens and keratins. Regarding protein composition, the proteomic analysis performed in previous work demonstrates an abundant collagen protein profile with about 31 collagen types (Stone et al., 2021). In line with this, we observed that collagen family proteins are the most abundant in MatriXpec™. The protocol applied by the manufacturer of the MatriXpec^TM^ hydrogel (Liguori et al., 2020) guarantees the complete decellularization of the native tissue, even though tissue processing has the negative bias of losing the molecular content of the extracellular matrix of interest. We do not detected remnants of nucleic acids into MatriXpec^TM^ hydrogel, either by extraction and Qubit assay for gDNA dosage or in fluorescence assays using DAPI, in line to other studies that postulate the absence of nucleic components as an indication of efficiency in the decellularization process (Ozlu et al., 2019).

We showed a high level of viability for hASCs seeded on MatriXpec™, in agreement with previous studies that demonstrated continuing cell viability on dECM-derived biomaterials (Chang et al., 2014; Sani et al., 2022). The findings from the LDH assay revealed that hASCs cultured on MatriXpec™ did not show significant differences in cell death compared to the culture performed on TCP, confirming the non-cytotoxic characteristics of the biomaterial. The qualitative assay employing markings for live and dead cells corroborated the LDH analyses, demonstrating that most cells plated on MatriXpec™ remain alive after 7 days of cell culture. We monitored cell cultures by counting nuclei and DNA dosage over time and showed that the growth of the MatriXpec™-plated hASCs is different from that observed in TCP-plated hASCs. Cells in the 3D culture microenvironment showed slower growth than in TCP experiments, as demonstrated by the lower number of cells after 7 and 14 days of cell culture. However, after 21 days of cell culture, we found no difference between the number of cells or DNA dosage in MatriXpec™ and TCP experiments. According to the literature, we hypothesized that the lower number of hASCs observed during the first 14 days of cell culture on MatriXpec™ hydrogel may be related to the new microenvironment challenges. Because they receive a myriad of three-dimensional information, their growth does not occur in an accelerated manner, as observed in cells plated above the TCP flat surface (Cavalcanti et al., 2013).

The morphology of hASCs plated on MatriXpec™ also showed remarkable changes compared to the TCP experiments over 21 days. Cell growth in a two-dimensional environment represented by TCP resulted in cell spreading, remodeled cell shape, and cytoskeleton organization. This remodeling is related to changes in the cell nucleus shape, which can lead to changes in gene expression and protein synthesis (Thomas et al., 1972). The 2D cell culture model does not represent the physiological environment in native tissue, with structures naturally organized in a 3D architecture. There is a demand for 3D culture approaches, which can recreate the native environment of cells and thereby allow them to maintain their three-dimensional shape and function, interacting with adjacent cells by receiving and transmitting signals and ultimately reducing stress and artificial responses that may be transmitted by cells in response to two-dimensional culture surfaces (Knight and Przyborski, 2015). In our study, when cultured on MatriXpec™, hASCs adopted a less sprawling and more elongated morphology with a fusiform shape. It should be noted that at the time of plating, hASCs were seeded on the MatriXpec™ hydrogel at the moment following its gelation. However, after 7 days of cell culture, it was possible to observe cells occupying different focal planes of the biomaterial. This observation became even more evident on days 14 and 21 of cell culture, with a greater number of cells immersed in the hydrogel, invading the interior of the gelified structure. Considering that MatriXpec™ presents a fibrillar microtopography full of three-dimensional cues when seeded on MatriXpec™, the hASCs adhere to the biomaterial and emit cell membrane protrusions for cell-matrix interaction. Thus, we hypothesized that the fusiform shape adopted by hASCs in the MatriXpec™ is partly due to their interaction and movement to migrate through the hydrogel.

Collagen proteins and GAG retention in MatriXpec™ may act as a chondrogenesis-promoting factor, based on previous studies reporting that GAGs such as chondroitin sulfate and aggrecan may have chondroinductive performance *in vitro* (Ingavle et al., 2013; Sutherland et al., 2015). In this study, we investigated the differentiation capacity of hASCs when cultured on MatriXpec™ for 10 or 21 days. Quantification of GAGs after 21 days of cell culture demonstrated that MatriXpec™ promotes GAG deposition by the hASCs, even in the absence of a chondrogenic induction medium. Comparing TCP and MatriXpec™ conditions, we observed an approximately twofold increase in the amount of GAG deposition by hASCs cultured on MatriXpec™, both in cultures that received the induction treatment and in the maintenance medium. We showed that the culture of hASCs on MatriXpec™ acts synergistically with the chemical induction treatment and may present an advantage to obtaining features of cartilaginous tissue, such as the presence of characteristic ECM molecules as GAGs. In addition, we conducted an RTqPCR analysis that revealed the expression of SOX9 significantly increased in MatriXpec™ experiments. SOX9 is the master regulator of chondrogenic differentiation, playing a central role in the formation and homeostasis of cartilaginous tissue (Mori-Akiyama et al., 2003; Akiyama et al., 2005; Almalki and Agrawal, 2016) by directly regulating genes involved in the synthesis and secretion of key ECM components, such as collagen proteins and glycosaminoglycan molecules (Bell et al., 1997; Ronique Lefebvre et al., 1997; Furumatsu et al., 2016). The increased expression of SOX9 in hASCs cultured on MatriXpec™ raised the possibility that the expression of genes directly regulated by SOX9, such as collagen type 2 (COL2) and aggrecan (ACAN), was also increased by MatriXpec™. However, RTqPCR analyses revealed no statistically significant difference in the presence of COL2 and ACAN between TCP and MatriXpec™ cultures. Building upon these observations, we hypothesized that hASCs differentiated into a fibrocartilage phenotype. Considering that MatriXpec™ hydrogel is obtained from decellularized pig tendon cartilage tissue, a fibrocartilaginous tissue (Hilborn and Bjursten, 2011), genes associated with the fibrotic phenotype could be upregulated in hASCs cultured on MatriXpec™, since the plating microenvironment could be acting to instruct or induce a fibrocartilage phenotype by hASCs. To address this issue, we investigated the expression of metalloproteinase-13 (MMP13), collagen type 10 (COL10), collagen type 1 (COL1), and fibromodulin (FMOD) in hASCs cultured on TCP or MatriXpec™, induced with the chemical differentiation factors. Interestingly, the expression of three markers (MMP13, COL10, and COL1) showed statistically significant upregulation in MatriXpec™ experiments associated with chondrogenic induction after 21 days of cell culture. Type 1 collagen protein is characteristic of the extracellular matrix of fibrocartilage tissues (Hellio Le Graverand et al., 2001), as well as COL10, which is produced by fibrocartilage cells near the tidemark between calcified and noncalcified cartilage layers (Niyibizi et al., 1996). In the case of tendon/ligament fibrocartilage, COL10 persists throughout maturity, which is different from its transient expression by hypertrophic chondrocytes in the hyaline cartilage of the growth plate (Benjamin and Ralphs, 1998; Yildirim et al., 2023).

In summary, we showed that the treatment for cartilage induction associated with MatriXpec™ culture acted synergistically, resulting in increased cartilage differentiation, as seen by the expression of SOX9 and GAG deposition. However, the hASCs plating on MatriXpec™ occupy and interpret signals from a microenvironment of fibrotic origin—tendon fibrocartilage—and thus start to respond to these signals by differentiating to a fibrocartilaginous phenotype. This study was limited by the high donor variability of hASCs. However, this is also advantageous, as it reflects the real clinic landscape.

We have shown the improved biological and physical properties of dECM MatriXpec™ hydrogel for 3D human adipose stem cell (hASCs) culture and fibrocartilage differentiation. The MatriXpec™ microtopography analysis revealed that this biomaterial has a fibrous ultrastructure composed mainly of collagen proteins. Cell culture on MatriXpec™ greatly maintained hASCs viability and growth. hASCs cultivated in MatriXpec™ adopted a spindle-shaped morphology in response to the three-dimensional growing microenvironment. Finally, the hASCs cultured on MatriXpec™ increase GAG deposition and expression of fibrocartilage-related genes.

## Data Availability

The mass spectrometry proteomics data have been deposited to the ProteomeXchange Consortium via the PRIDE partner repository with the dataset identifier PXD047464.
