# Supplementary material for: Bioactive decellularized extracellular matrix-based hydrogel supports human adipose tissue-derived stem cell maintenance and fibrocartilage phenotype
Source: Front Bioeng Biotechnol. 2024 Jan 8;11:1304030. doi: 10.3389/fbioe.2023.1304030 (PMC10800544; doi:10.3389/fbioe.2023.1304030)
Supplement: Supplementary file 1 [file Image1.PDF]

**Bioactive decellularized extracellular matrix-based hydrogel supports human adipose tissue-derived stem cell maintenance and fibrocartilage phenotype**

**Supplementary information**

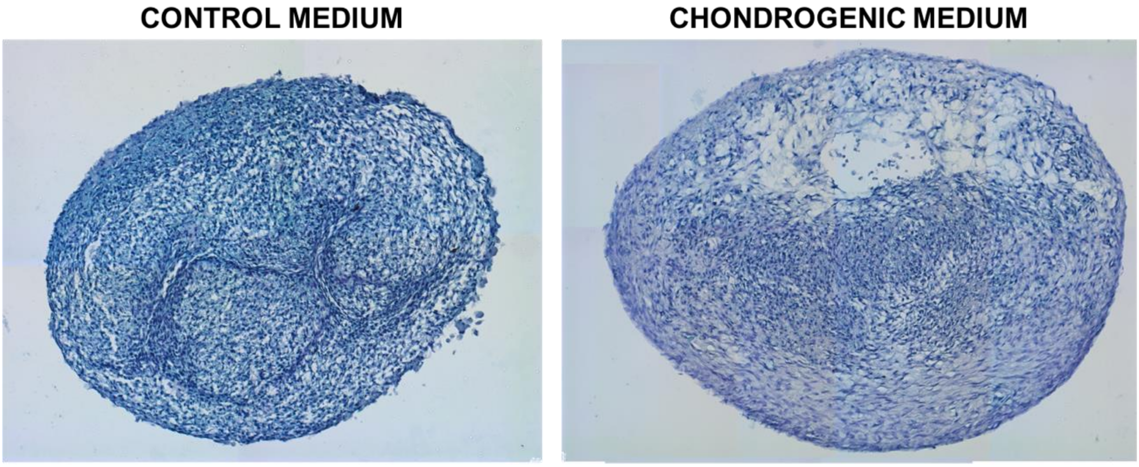


**Figure S1. Micromass culture of hASCs and chondrogenic differentiation induction.** Chondrogenic differentiation from hASCs cultured on micromass was evaluated by alcian blue staining. For tube-based micromass cell culture, hASCs were seeded at a density of $1x10^6$ cells/tube in 15 ml centrifuge tubes. Tubes were centrifuged at 400 g for 10 minutes to accelerate the formation of inicial cell micromasses. hASCs were cultivated in Dulbbecco's modified Eagle's medium, 4 mM L-glutamine, 100 U/mL penicillin and 100 ug/mL streptomycin at 37˚C with 95% humidity and 5% CO2. Experiments were conducted on the cells between passages 4 and 6. The micromasses were induced for chondrogenic differentiation for a period of 21 days following the same protocol described in section 2.7 of Materials and Methods. Cartilage differentiation of the hASCs after 21 days of culture was assessed by alcian blue staining through histological analysis.
